# Evaluation of laser-assisted surgery in minimally invasive deciduous teeth extraction

**DOI:** 10.6026/973206300213592

**Published:** 2025-10-31

**Authors:** Muhammed Riyas Ali, Beena George, Vanita Mansukhani, Sangeetha J. Kadavan, Sharon Vincent, Pooja Bhagwat

**Affiliations:** 1 Department of Oral and Maxillofacial Surgeon, Pathiripala, Palakkad, Kerala, India; 2 Department of Oral Pathology and Microbiology, Sree Mookambika Institute of Dental Sciences, Kulasekharam, Tamil Nadu, India; 3 Deaprtment of Conservative Dentistry and Endodontics, Sree Balaji Dental College, Velachery Rd, Narayanapuram, Pallikaranai, Chennai, Tamil Nadu, India; 4 Department of Pedodontics and Preventive Dentistry, P.S.M. College of Dental Science and Research Bypass Road, Akkikavu, Thrissur, Kerala, India; 5 Department of Pediatric and Preventive Dentistry, Al Azhar Dental College, Thodupuzha, Kerala, India; 6 Department of Oral Pathology and Microbiology, VYWS Dental College and Hospital SRPF, Tapovan Vidyapeeth Road Camp, Amravati, Maharashtra, India

**Keywords:** Laser dentistry, pediatric dentistry, deciduous tooth extraction, diode laser, minimally invasive surgery, postoperative pain, healing

## Abstract

Laser-assisted techniques are gaining popularity in pediatric dentistry due to reduced pain and faster healing. This study compared laser-assisted and conventional extractions in 60 children needing deciduous tooth removal. Pain levels, procedure time and healing were the main outcomes analyzed. Laser-assisted methods showed significantly less pain and quicker recovery. Thus, we show further research into laser techniques to enhance comfort and compliance in children.

## Background:

Minimally invasive dentistry has the goal of limiting trauma for patients undergoing dental procedures. In children, trauma should be viewed as a combination of the anxiety of what can happen and the pain that a child has tolerance for [1]. Extraction of a primary tooth is common and typically performed while using local anesthesia, yet analgesia, loading, bleeding, swelling and/or infection [2]. This is where the use of laser technology can improve the patient experience and surgical precision [3]. Lasers, both diode and CO_2_ lasers, provided a host of advantages including hemostasis, bactericidal effects possibly limited postoperative pain [4]. In children, who have a generally lower pain tolerance as well as higher dental-related fear, these advantages can be useful when practicing dentistry [5]. Previous research has indicated successful outcomes of using lasers for surgeries dating back to 1999 using lasers for soft-tissue surgeries and frenectomies [6]. Recent trials and clinical reports suggest that laser-assisted tooth extraction of both primary and permanent teeth improve visibility in the surgical field, success and minimize tissue damage for superior wound healing [7]. Additionally, studies have not performed randomized studies comparing clinical outcomes, such as allergic reaction, between laser-assisted versus traditional methods in performing deciduous tooth extractions [8]. Therefore, it is of interest to report on the outcomes of laser-assisted extraction of deciduous teeth in children, with particular emphasis on its clinical efficacy, procedural efficiency and impact on post-operative comfort.

## Materials and Methods:

This prospective, case-controlled study was completed in the Pediatric Dental Unit of a tertiary care teaching hospital. A total of 60 pediatric patients aged between 5 to 9 years impacted or requiring extraction of at least one deciduous tooth were included. Patients were randomly assigned into two groups: Group A (n=30) underwent laser-assisted extraction with laser-assisted a 980 nm diode laser and Group B (n=30) received conventional extraction with dental forceps and elevators with local anesthesia. Inclusion criteria included children requiring extraction due to physiological exfoliation, dental caries, or orthodontic indications. Exclusion criteria were systemic illnesses, uncooperative behavior and infection at the extraction site. All procedures were performed by a single operator to eliminate technique variability. Intraoperative time, bleeding, pain (using Wong-Baker FACES scale) and postoperative healing were recorded. Follow-up was done on day 1, day 3 and day 7 post-extraction.

## Results:

A total of 60 pediatric patients aged 5-9 years were enrolled, equally divided into two groups: Group A (laser-assisted extraction, n=30) and Group B (conventional extraction, n=30). Baseline characteristics, including mean age, gender distribution and number of teeth extracted, were comparable, confirming that outcome differences were not due to demographic imbalance. Intraoperative assessment demonstrated that laser-assisted procedures were associated with shorter operative time, markedly less bleeding and lowers immediate pain scores. Postoperative evaluation on day 1 showed that children treated with lasers experienced significantly reduced pain and swelling compared with those who underwent conventional extractions. By day 7, healing was more favorable in the laser group, with a higher rate of complete healing and reduced need for analgesics. Collectively, these findings indicate that laser-assisted extraction provides substantial benefits in terms of efficiency, comfort and recovery in pediatric dental patients. Table 1 (see PDF) confirmed that both groups were comparable in baseline demographics and number of extractions. Table 2 (see PDF) demonstrated that laser-assisted extractions were faster, less invasive and associated with lower intraoperative pain and bleeding. Table 3 (see PDF) showed that postoperative pain and swelling were significantly reduced in the laser group by day 1. Table 4 (see PDF) indicated superior healing outcomes in the laser group, with fewer children requiring analgesics and a higher rate of complete healing at day 7. Collectively, these results reinforce the clinical efficacy and patient comfort advantages of laser-assisted surgery for deciduous tooth extraction.

## Discussion:

The use of laser-assisted surgical removal of primary dentition affords a number of important distinct clinical benefits for children. The results of this study demonstrated significant advantages in terms of shorter procedural time, reduced bleeding, lower pain scores and faster healing in children treated with diode lasers [9]. These results are echoed in previous studies which have examined lasers with regard to the hemostatic effects and pain reduction [10]. Intraoperative pain reduction can be largely attributed to the photothermal effect of the diode laser, plugs the nerve endings and blood vessels [11]. Additionally, the laser cuts and cauterizes, causing less trauma to the tissues and reducing swelling postoperatively [12]. The healing in the laser group was markedly improved and is supported in the literature in studies of frenectomy and gingivectomy in children [13]. Laser utilization in pediatric dentistry is becoming more widely accepted and usable, partly due to improved safety profiles and the increasing availability of affordable portable devices [14]. While diode lasers definitely have a role to play in pediatric dentistry because they are inexpensive and ease of use, other newer technologies such as erbium or CO_2_ lasers may plainly do more, especially situations of hard tissue use [15]. Nonetheless, there are inherent limitations to this new technology. The learning curve to utilize laser procedures, as well as capital costs of laser devices may inhibit universal acceptance of the utility [16]. Additional studies are necessary to determine if there are any long-term impact of laser extraction on tooth eruption timing and adjacent structures [17].

## Conclusion:

Laser-assisted extraction of primary teeth in pediatric patients greatly improves intraoperative comfort during and after surgery and improves healing time. Using diode lasers in routine pediatric dentistry can potentially enhance patient cooperation during treatment and promote better treatment outcomes. Further randomized multi-center clinical trials are recommended to confirm the sustained benefits.
